# Absence of Parkin Results in Atrophy of Oxidative Myofibers and Modulation of AKT and MURF1 Signaling in Middle‐Aged Male Mice

**DOI:** 10.1111/apha.70082

**Published:** 2025-07-29

**Authors:** Isabela Aparecida Divino, Ana Laura da Vieira‐da‐Silva, Marcos Vinicius Esteca, Rafael Paschini Tonon, Felipe Oliveira Gomes da Cruz, Renata Rosseto Braga, Eduardo Rochete Ropelle, Paulo Guimarães Gandra, Igor Luchini Baptista

**Affiliations:** ^1^ Laboratory of Cell and Tissue Biology (LaBCeT), School of Applied Sciences University of Campinas Limeira Brazil; ^2^ Laboratory of Molecular Biology of Exercise (LaBMEx), School of Applied Sciences University of Campinas Limeira Brazil; ^3^ Skeletal Muscle Biology Laboratory, Department of Biochemistry and Tissue Biology, Institute of Biology University of Campinas Campinas Brazil

**Keywords:** aging, E3 ubiquitin ligase, muscle loss, protein synthesis signaling, skeletal muscle

## Abstract

**Aim:**

This work aimed to investigate the effects of the loss of Parkin in middle‐aged mice skeletal muscle, focusing on different types of myofibers and in the analysis of proteins related to protein synthesis and degradation as well as the analysis of force generation and motor balance.

**Methods:**

We used male mice C57BL/6J (WT) and Parkin knockout mice, Parkintm1Shn (Parkin^−/−^) at 3 and 10 months of age. We used Walking Beam, Open Field, Spider Mice and Maximum Power Tests to assess motor, balance, and endurance functions. We used flexor digitorum brevis (FDB) muscle for force generation analysis, and tibial anterior (TA) and soleus (SOL) muscles were used for biomolecular techniques because of their difference in fiber type. These muscles were used to investigate markers of protein synthesis and degradation, mitochondrial respiration, and myofiber diameter.

**Results:**

The Absence of Parkin in middle‐aged mice leads to a reduction in isometric force generation but maintained overall motor and locomotion abilities, exhibited only minor balance deficits. In the SOL muscle of middle‐aged Parkin^−/−^ mice, we observed a reduction of muscle mass and myofiber diameter, also a significant decrease in mitochondrial respiratory capacity and Complex V. In the same group, we observed a reduction in the phosphorylation of AKT and 4E‐BP1, and an increase in *MURF‐1* while Ubiquitin K63 levels decreased. We did not observe relevant differences in the TA muscle.

**Conclusion:**

Our results suggest middle‐aged Parkin^−/−^ mice exhibited muscle atrophy and mitochondrial dysfunction primarily in oxidative myofibers before noticeable motor dysfunction occurs.

## 1 Introduction

Skeletal muscle tissue is a regulatory organ with essential functions such as control of glucose metabolism, amino acid reserve, and endocrine secretion. The loss of muscle mass is associated with neurodegenerative diseases like Parkinson's [1]. In Parkinson's disease, losing dopaminergic neurons impacts motor locomotion, balance, and muscle strength, resulting in subsequent muscle atrophy [2]. Individuals with Parkinson's disease exhibit histopathological abnormalities in skeletal muscle, characterized by necrosis, inflammation, and dysfunction in the mitochondrial respiratory chain complexes [3]. Additionally, the loss of muscle mass resulting from the disease may be related to the fiber type, in which individuals with Parkinson's disease exhibit an accumulation of type I myofiber [4]. However, despite the comprehensive pathological content in the literature, the mechanisms through which skeletal muscle may be affected before the onset of motor symptoms and subsequent muscular atrophy remain uncertain.

Genetic mutations contribute to approximately 10% of Parkinson's disease cases, with over 10 identified genes, including the *PARK2* gene. Mutations in *PARK2* (also referred to as PRKN/PARKIN) lead to the second best‐known form of familial Parkinson's disease, accounting for nearly 50% of cases in its juvenile autosomal recessive form [5]. Parkin is a 465‐amino acid E3 ubiquitin ligase that plays an essential role in maintaining mitochondrial integrity through mitophagy. Parkin‐dependent mitophagy occurs through the activation of the protein by PINK1 [6]. Parkin ubiquitinates outer mitochondrial membrane proteins such as voltage‐dependent anion channels (VDACs) and translocase of outer mitochondrial membrane 20 (TOMM20) to initiate mitophagy. The ubiquitin chains left by Parkin are recognized by autophagic receptors (p62/SQSTM1), which form the autophagosome membrane upon binding to LC3 I and II. Finally, the mitochondria are degraded by the fusion of the autophagosome with the lysosome [7, 8, 9].

Although Parkin knockout mice (Parkin^−/−^) may not precisely replicate Parkinson's disease in humans, studies have demonstrated that this model tends to anticipate phenotypes associated with the loss of dopaminergic neurons and compromised mitochondrial quality, which are underlying factors in Parkinson's disease [10, 11]. Parkin plays a key role in regulating muscle mass and mitochondrial function in skeletal muscle tissue, and its deficiency in mice leads to muscle atrophy and impaired muscle contraction [12, 13]. One study showed that the loss of Parkin exacerbates fasting‐induced muscle loss in mice by positively regulating catabolic genes [14]. Another study found that overexpression of Parkin in the tibial muscle (TA) of aged mice attenuates age‐related muscle loss, improves mitochondrial enzyme activities, and increases strength [15]. Additionally, our group demonstrated that Parkin^−/−^ mice exhibit impaired muscle regeneration after injury to the anterior TA muscle [16].

Indeed, it is evident that Parkin plays a fundamental role in maintaining skeletal muscle health, and its loss or dysfunction leads to tissue disorders. However, there is limited knowledge regarding the impact of Parkin deficiency on skeletal muscle in middle age. Thus, we focused on analyzing the skeletal muscle of 10‐month‐old Parkin^−/−^ mice, particularly emphasizing mitochondrial function and protein synthesis in two muscles with different myofiber type prevalence. Our findings demonstrate that middle‐aged Parkin^−/−^ mice exhibit a reduced diameter of oxidative myofibers, decreased activation of proteins associated with protein synthesis, and increased expression of genes linked to proteasomal degradation. Notably, these alterations precede motor impairment, providing new insights into the role of Parkin in skeletal muscle tissue.

## 2 Methods

### 2.1 Animal Models

The mice used in this study were male C57BL/6J mice (WT) and Parkin knockout from the Parkin^tm^1Shn lineage (Parkin^−/−^). Parkin knockout mice were generated by Goldberg et al., 2003, and provided by The Jackson Laboratory. Both genotypes were kept by the Multidisciplinary Center for Biological Research in Laboratory Animal Science, CEMIB. Ethics Committee N° 5628‐1/2020. During the experiments, the animals were kept at the Central Biotery of the Faculty of Applied Sciences, where the humidity was monitored between 40% and 60%. We kept the mice in a 12‐h light/dark cycle in cages arranged in cabinets with air exhaust, with a temperature ranging from 20°C to 26°C. The feed used for nutrition was standard commercial feed (Nuvilab), and drinking water was provided *ad libitum*. The study followed the mice from 3 to 10 months of age. A total of 80 mice were used in this study. Euthanasia was performed by anesthetic overdose using 30 mg/kg xylazine and 300 mg/kg ketamine. After euthanasia, the tibial anterior (TA), soleus (SOL), and flexor digitorum brevis (FDB) muscles were collected for analysis.

### 2.2 Walking Beam Test

We adapted the test by Luong TN et al. [17]. Mice were trained for two consecutive days to cross three beams of different diameters (6, 12, and 18 mm) and 80 cm long. On test day, we used a camera positioned to capture the start and end of each beam. The mice traversed each beam once, starting from the largest diameter to the smallest. The test was performed during the light cycle. The measurements were the number of slips with the hind paw, the number of footprints, beam crossing time, and the number of stimuli given so the mice could follow the path when necessary.

### 2.3 Open Field

The protocol was adapted from Walsh and Cummins [18]. Mice were habituated for two consecutive days before testing. The open field consists of a circular arena 60 cm in diameter and walls 48 cm high, the upper part being open and the lower part containing the limit of 19 quadrants. The mice remained in the arena for 10 min, with 5 min for recognition and adaptation to the location, and the final 5 min were recorded using a camera (Canon t2i). The test was conducted between 6 and 8 a.m. in the Faculty of Applied Sciences vivarium, where the environment regarding noise and odors was controlled. The camera was placed on a support so that the lens was directed perpendicularly to the lower part of the arena. Peripheral ambulation (peripheral distance traveled) and total ambulation (total distance covered) were analyzed. We evaluated these parameters using the ToxTrac software, and an analyst blindly analyzed the videos.

### 2.4 Power Test

The mice were previously adapted to a treadmill (AVS Projetos) the day before the test. The test consisted of determining the maximum running power of the animals. The mice were placed on the treadmill, which increased speed every 3 min. The initial speed was 12 m per minute. The experimenter manually stimulated the mice to encourage continued activity, and fatigue was determined when they no longer responded to the stimulation. Subsequently, they were removed from the treadmill, and travel time, speed, and distance were recorded. The test was performed during the light cycle.

### 2.5 Spider Mice

The test was conducted at 10:00 a.m. We previously adapted the mice to a grid measuring 45 cm × 45 cm, with 1 cm × 1 cm squares, where the mice's grip would hang suspended above a water drum. The adaptation involved letting them hang and directing them into the water three times. The test began after adaptation, and the mice were placed on the grid individually. After that, a water drum was placed under the grid. Then, the animals were hanging upside down, and the time they remained hanging upside down until they fell into the water was recorded. All mice underwent the test three times, and the average times were used.

### 2.6 Single Fiber Dissection and Preparation

Force generation was measured in living, isolated single skeletal muscle fibers. Intact single fibers with tendons were mechanically dissected from the FDB muscles of WT and Parkin^−/−^ mice. Dissection was carried out in a solution containing (in mM): 136 NaCl, 5 KCl, 1.8 CaCl_2_, 0.5 MgCl_2_, 0.4 NaH_2_PO_4_, 11.9 NaHCO_3_, and 5.5 glucose [19]. Each fiber was mounted on a Small Intact Muscle Apparatus (model no. 801C, Aurora Scientific Inc., Aurora, ON, Canada). The fiber tendons were secured to aluminum T‐clips, which were connected by kooks to a force transducer (model no. 403A, Aurora Scientific Inc. Aurora Scientific Inc.) on one end and to a micropositioner on the opposite end. During the experiments, fibers were continuously perfused with Tyrode solution (composition in mM: 121 NaCl, 5 KCl, 1.8 CaCl_2_, 0.5 MgCl_2_, 0.4 NaH_2_PO_4_, 24 NaHCO_3_, 5.5 glucose, and 0.1 EDTA), which was bubbled with 95% O_2_ and 5% CO_2_, to maintain the pH at ~7.4, at a temperature of ~28°C (which is close to the temperature of the FDB muscle in vivo) [20]. Electrical stimulations were evoked via parallel platinum electrodes using a Grass S88X stimulator (Grass Technologies, Quincy, MA, USA). Stimulation parameters were set to 350 ms train duration, with 0.5 ms biphasic pulses at 8 V. The fiber length was adjusted to achieve the maximal isometric tetanic tension.

### 2.7 Isometric Force Measurements and Experimental Protocol

Each fiber was stimulated at pulse frequencies ranging from 1 to 120 Hz to obtain a force–frequency curve (using 350 ms train duration, 0.5 ms biphasic pulses at 8 V). Contractions were spaced with 1 min intervals. A total of 5 fibers from 3 different WT mice and 5 fibers from 3 different Parkin^−/−^ mice were analyzed. After a 10 min resting period, 3 fibers from WT mice, and 4 fibers from Parkin^−/−^ mice underwent a stimulation protocol to evaluate fatigue resistance. The fatigue protocol consisted of repetitive contractions induced by 70 Hz stimulations at an initial train frequency of 0.25 trains/s that was increased every 2 min to 0.33, 0.5, and 1.0 trains/s as previously described [21]. The protocol was terminated when peak force generation declined to 50% of the first contraction. Data were processed and analyzed using Dynamic Muscle Analysis Software version 6.000 (Aurora Scientific Inc., Aurora, ON, Canada).

### 2.8 Hematoxylin and Eosin Staining

Serial sections of the TA were obtained with a thickness of 9 μm using a cryostat. The staining procedure was as follows: 95% alcohol for tissue fixation for 2 min. The alcohol was removed, hematoxylin was added for 90 s, followed by eosin 0.5% for 1 min. Afterward, the slides were subjected to 3‐min baths in 70%, 80%, and 95% alcohol, respectively. Absolute alcohol was then used for three 3‐min baths and three washes with xylene. Once dry, the slides were coverslipped and mounted with Entellan. The images were acquired by light microscope (Leica) using a 20× and 40× objective. The analysis was done double‐blinded, and 500 fibers for TA and 300 fibers for SOL were measured using *ImageJ* based on Ferret's measurement.

### 2.9 Myosin ATPase Activity

The SOL muscle fiber types (I and II) were measured using the myosin ATPase detection technique described in Survana et al. [22]. After preservation, the tissue was sectioned into 8 μm slices. For each animal, two slides were used: one for glycine buffer (pH 9.4) and acid buffer (pH 4.6) staining. All images were acquired by light microscope (Leica) using a 20× and 40× objective. The results were measured blindly using *ImageJ* based on Ferret's measurement, and 250 fibers were analyzed.

### 2.10 High‐Resolution Respirometry

Mitochondrial respiration was assessed by high‐resolution respirometry using the Oxygraph‐2 k (Oroboros Instruments, Innsbruck, Austria). A 20 mg of the dissected TA and 5 mg of SOL muscles were permeabilized by manual fiber separation, followed by a chemical permeabilization with saponin (50 ug). The tissues were added to the respiratory chamber containing 2 mL of MIR05 buffer (110 mM sucrose, 60 mM K‐lactobionate, 0.5 mM EGTA, 3 mM MgCl_2_, 20 mM taurine, 10 mM KH2PO4, 20 mM HEPES, pH 7.1, and 0.1% BSA). Measurements were performed at 37°C. To measure mitochondrial complex I‐dependent respiration, malate (0.5 mM) and glutamate (10 mM) were added to the chambers. To measure respiratory stimulation, ADP (2.5 mM) was added. Combined complex I and complex II respiration was assessed by the addition of succinate (10 mM). Subsequently, mitochondrial coupling was evaluated by the inhibition of ATP synthase by adding oligomycin (2.5 μM). The maximal capacity of the electron transport system was measured by a multiple‐step carbonylcyanide p‐trifluoromethoxyphenyl‐hydrazone (FCCP) (0.5 μM) titration. After each addition, a plateau in oxygen consumption was awaited.

### 2.11 Western Blot

The SOL and TA muscles were homogenized and diluted in extraction buffer (0.625% Nonidet P‐40, 0.625% [W/V] sodium deoxycholate; 0.00625 M sodium phosphate pH 7.2, 1 mM EDTA pH 8, and 1% phosphatase and protease inhibitors) and centrifuged at 15 000 G for 10 min. The supernatant was collected and quantified using the Bradford method (Bio‐Rad). The SDS‐PAGE gel was prepared with a 4%–20% gradient, and a 5% stacking gel was used. 40 μg of protein diluted in the Laemmli buffer was applied for electrophoresis. Before incubation with the primary antibody, the membrane underwent four washes of 5 min each with TBS‐T (0.1% Tween 20) and was pre‐blocked with a 5% BSA solution in TBS‐T for 1 h. The primary antibodies were diluted in a solution containing 5% BSA in TBS‐T and incubated overnight at 4°C. After incubation with the primary antibody, the membrane was rewashed 4 times with TBS‐T and incubated with peroxidase‐conjugated secondary antibodies at a 1:10 000 ratio in TBS‐T solution. Finally, the membrane was washed three times and revealed using the Luminata Forte Western HRP reagent (EMD Millipore) and a photo documenter. Primary antibodies used included: p‐4E‐BP1 (Thr 70)—Cell Signaling, #9455s, 1:1000; 4E‐BP1—Cell Signaling #9452s, 1:1000; AKT—Santa Cruz #sc‐8312, 1:500; p‐AKT (Ser 473)—Santa Cruz #sc‐33437, 1:500; PGC‐1α—Invitrogen #Pa5‐38022, 1:1000; Ubiquitin—Invitrogen #PA1‐10023, 1:1000; Ubiquitin K48—Invitrogen #MA5‐35382, 1:1000; Ubiquitin K63—Invitrogen #MA5‐32573, 1:1000; TOM20—Cell Signaling #42406s, 1:1000; LC3A/B—Cell Signaling #4108s, 1:1000; SQSTM1/p62—Cell Signaling #23214s, 1:1000; OXPHOS—Abcam #ab110413.

### 2.12 Real‐Time PCR

RNA extraction was performed using 1 mL of Trizol on previously extracted and stored tissues (Invitrogen, 15596026), following the manufacturer's instructions. Purity and quantification were evaluated by spectrophotometry (Epoch BioTek, Winooski, VT, USA). Reverse transcription was conducted with 500 μg of isolated RNA. Subsequently, gene expression was assessed through fluorescence quantification utilizing the 7500 Fast Real‐Time System (Applied Biosystems; Thermo Fisher Scientific Inc.), applying the 2−ΔΔCt formula as outlined by K. Livak in the Biosystem Sequence Detector Bulletin 2. The primers used for qPCR are listed below:

**Table:** 

Gene	Forward	Reverse
MuRF‐1	GTGTGAGGTGCCTACTTGCT	ACTCAGCTCCTCCTTCACCT
Mafbx (Atrogin 1)	TACTAAGGAGCGCCATGGATACT	GTTGAATCCTCTGGAATCCAGGAT
GAPDH	CATCACTGCCACCCAGAAGACTG	ATGCCAGTGAGCTTCCCGTTCAG

### 2.13 Statistical Analyses

We used Student's *t*‐test to analyze differences between two groups and One‐Way ANOVA with Bonferroni post‐test to analyze differences between four groups. In our analysis of differences in force generation between the two groups, we performed a two‐way ANOVA followed by a Bonferroni post‐test. The Kolmogorov–Smirnov test was applied to assess adherence to normality. The significance level was set at *p* < 0.05. All statistical analyses were performed using GraphPad Prism 8.4.3 software.

## 3 Results

### 3.1 Parkin^−/−^ Middle‐Aged Mice Do Not Exhibit Relevant Motor Impairments

**FIGURE 1:**
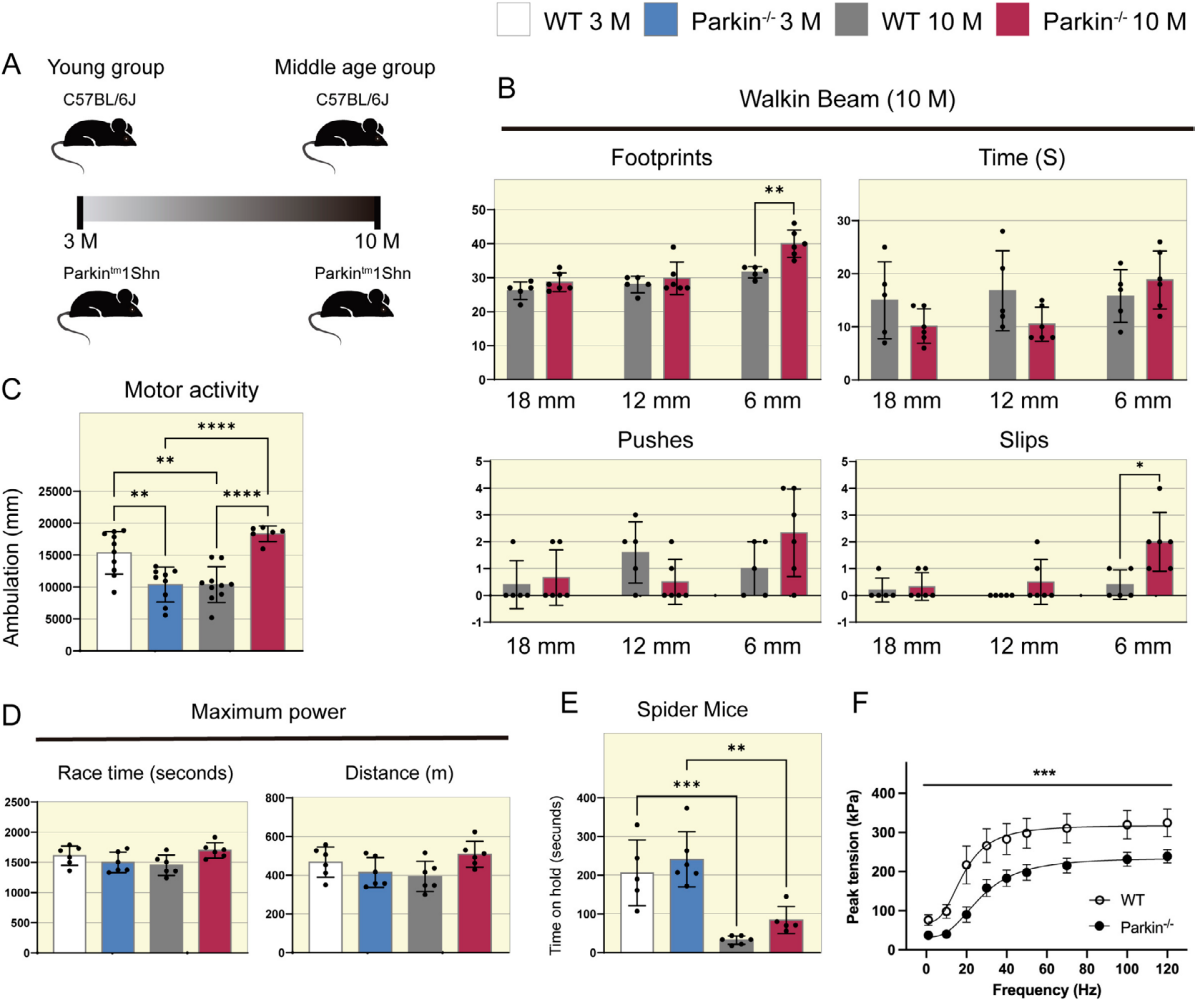
Motor capacity and balance in WT and Parkin^−/−^ mice at ages 3 and 10 months. (A) Experimental design. (B) Walking Beam Test of WT and Parkin^−/−^ mice at 10 months. The graphics demonstrate the analysis of footprints, walking time, pushes, and slips (*n* = 5–6). Data are presented as means ± SD. Student's *t*‐test: *p < 0.05, **p < 0.01. WT 10 M is set as 1. (C) Open Field Test of WT and Parkin^−/−^ mice at ages 3 and 10 months. The graphic demonstrates the analysis of total ambulation (*n* = 6–10). Data are presented as means ± SD. One‐way ANOVA followed by Bonferroni's post hoc test: **p < 0.01, ****p < 0.0001. WT 3 M is set as 1. (D) Maximum Power Test of WT and Parkin^−/−^ mice at ages 3 and 10 months. The graphics demonstrate the measurement of maximum time spent on the treadmill and distance covered (*n* = 6). Data are presented as means ± SD. WT 3 M is set as 1. (E) Spider Mice Test of WT and Parkin^−/−^ mice at ages 3 and 10 months. The graphic demonstrates the grasping time (*n* = 5–6). Data are presented as means ± SD. One‐way ANOVA followed by Bonferroni's post hoc test: **p < 0.01, ***p < 0.001. WT 3 M is set as 1. (F) Force versus stimulation frequency data from FDB muscle fibers from WT and Parkin^−/−^ mice (*n* = 5 fibers from 3 WT mice and *n* = 5 fibers from 3 and Parkin^−/−^ mice). Data are presented as means ± SEM. Two‐way ANOVA followed by Bonferroni's post hoc test: ***p < 0.001.

Mice were divided into four groups, WT and Parkin^−/−^, at ages 3 and 10 months (Figure 1A). Firstly, we aimed to analyze the mice's motor functionality. We conducted the Walking Beam Test to assess if middle‐aged Parkin^−/−^ mice had deficits in balance. We did not find significant differences in the number of stimuli or beam‐crossing time between the groups (Figure 1B). However, in the smaller diameter beam (6 mm), Parkin^−/−^ mice displayed more footprints (WT 10 M 32 vs. Parkin^−/−^ 10 M 40 footprints, Figure 1B) and more slips (WT 10 M 0.5 vs. Parkin^−/−^ 10 M 2 footprints, Figure 1B) compared to WT mice, which may indicate locomotor adaptations in response to a challenge (narrow beam).

Next, we performed the Open Field Test to evaluate locomotor activity, exploration habits, and anxiety levels. At 3 months of age, Parkin^−/−^ mice exhibit less total ambulation than WT mice (WT 3 M 15 000 vs. Parkin^−/−^ 3 M 10 000 mm, Figure 1C) in the same age. In middle age, WT mice have reduced ambulation compared to young WT mice (WT 3 M 15 000 vs. WT 10 M 10 000 mm, Figure 1C). In contrast, the Parkin^−/−^ genotype has increased total ambulation in middle age compared to young Parkin^−/−^ mice (Parkin^−/−^ 3 M 10 000 vs. Parkin^−/−^ 10 M 18 000 mm, Figure 1C) and compared to WT mice at the same age (WT 10 M 10 000 vs. Parkin^−/−^ 10 M 18 000 mm, Figure 1C). We also observed that Parkin^−/−^ mice in middle age have increased ambulation in the central area of the Open Field compared to the same younger genotype (Parkin^−/−^ 3 M 7% vs. Parkin^−/−^ 10 M 14%, Figure S1A). Lastly, we used two tests, Maximal Power on a treadmill and Spider Mice, to assess motor performance. In the first test, we found no differences between the groups (Figure 1D). In the second test, both genotypes at 10 months of age spent less time hanging on the grid than the younger groups (WT 3 M 200 vs. WT 10 M 30 s, Figure 1E) and (Parkin^−/−^ 3 M 220 vs. Parkin^−/−^ 10 M 80 s, Figure 1E).

### 3.2 Force Generation Is Decreased in Single Fibers From Parkin^−/−^ Middle‐Aged Mice

To investigate the role of Parkin in skeletal muscle contractility, we quantified specific force generation in isolated, intact single fibers from the FDB muscle of middle‐aged WT and Parkin^−/−^ mice. Maximal isometric force generation was significantly lower in fibers from Parkin^−/−^ mice compared to WT mice (WT 10 M 325 ± 17 kPa vs. Parkin^−/−^ 10 M 239 ± 35 kPa, Figure 1F). Similarly, submaximal force development evoked by different stimulation frequencies was reduced in fibers from Parkin^−/−^ mice compared to WT (Figure 1F). During the fatigue protocol, the time to reach a 50% decline in peak force was 213.5 ± 8.0 s for fibers from Parkin^−/−^ mice and 290.3 ± 32.6 s for fibers from WT (Figure S1B).

### 3.3 Oxidative Myofibers in Middle‐Aged Parkin^−/−^ Mice Exhibit Smaller Diameters Than WT Mice

**FIGURE 2:**
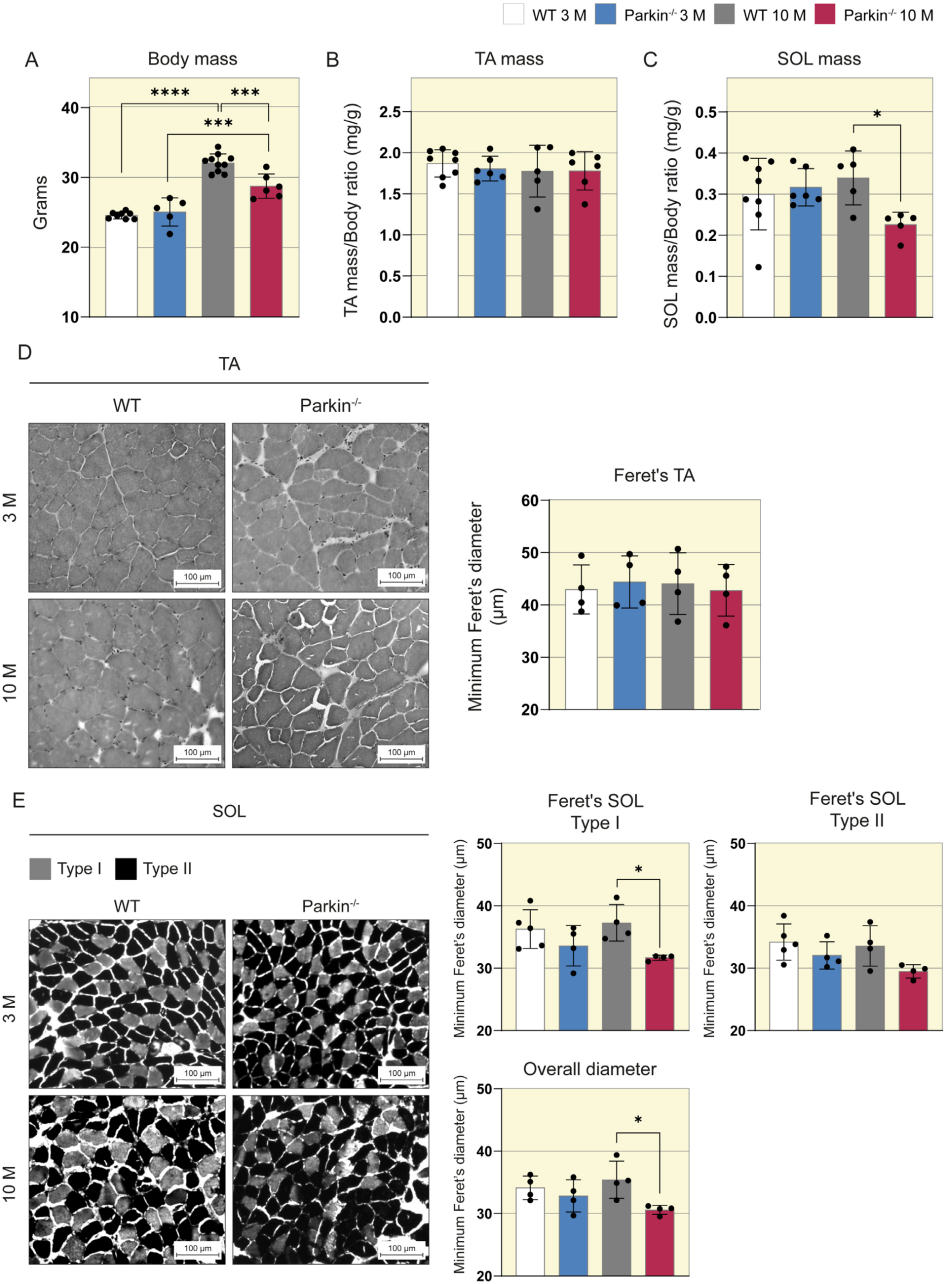
Diameters of muscle myofibers in WT and Parkin^−/−^ mice at ages 3 and 10 months. (A) The graphic demonstrates the body mass of WT and Parkin^−/−^ mice at ages 3 and 10 months (*n* = 5–10). Data are presented as means ± SD. One‐way ANOVA followed by Bonferroni's post hoc test: *p < 0.05, ***p < 0.01, ****p < 0.0001. WT 3 M is set as 1. (B) The graphic demonstrates the ratio of TA muscle mass to body mass of WT and Parkin^−/−^ mice at ages 3 and 10 months (*n* = 5–8). Data are presented as means ± SD. WT 3 M is set as 1. (C) The graphic demonstrates the ratio of SOL muscle mass to body mass of WT and Parkin^−/−^ mice at ages 3 and 10 months (*n* = 5–8). Data are presented as means ± SD. One‐way ANOVA followed by Bonferroni's post hoc test: *p < 0.05. WT 3 M is set as 1. (D) Representative images of H&E‐stained cross‐sections of TA muscle from WT and Parkin^−/−^ mice at ages 3 and 10 months, along with the corresponding graph showing the distribution of minimum Feret's diameter (*n* = 4). Scale bar: 100 μm. Data are presented as means ± SD. WT 3 M is set as 1. (E) Representative images of Myosin ATPase activity cross‐sectional area by fiber type of SOL muscle from WT and Parkin^−/−^ mice at ages 3 and 10 months, and the corresponding graphs showing the distribution of minimum Feret's diameter in type I, type II, and overall diameter of myofibers (*n* = 4–5). Scale bar: 100 μm. Data are presented as means ± SD. One‐way ANOVA followed by Bonferroni's post hoc test: *p < 0.05. WT 3 M is set as 1.

Whereas the loss of Parkin does not imply motor impairments until middleage, but results in impairment in maximal isometric force generation, we aim to analyze skeletal muscle mass and related pathways. Since the specific force and contractility of muscle can vary depending on the fiber type, and FDB muscle is predominantly composed of type II fibers, we decided to investigate the morphological parameters of the TA muscle and the SOL muscle, a well‐described muscle with a high percentage of type I fibers. We investigate muscle mass and myofiber diameter in both muscles. Initially, we analyzed body weight and observed that both genotypes gained body weight in middle age (Figure 2A). The TA muscle mass showed no differences (Figure 2B), but the SOL muscle mass in Parkin^−/−^ mice was smaller at 10 months compared to WT mice at the same age (WT 10 M 0.33 vs. Parkin^−/−^ 10 M 0.23 mg/g, Figure 2C). We also evaluated the Feret's diameter. We did not observe differences in the TA muscle between the groups (Figure 2D). However, the SOL muscle in Parkin^−/−^ mice showed a reduction of diameter in 10 months, both overall (WT 10 M 36 vs. Parkin^−/−^ 10 M 31 μm, Figure 2E) and in type I (WT 10 M 38 vs Parkin^−/−^ 10 M 32 μm, Figure 2E) fibers compared to middle‐aged WT mice. Our findings show that the loss of Parkin in middle‐aged mice leads to specific atrophy of oxidative myofibers, which occurs before motor impairments.

### 3.4 Soleus and Tibial Muscles of Parkin^−/−^ Mice Exhibit Differences in Protein Synthesis

**FIGURE 3:**
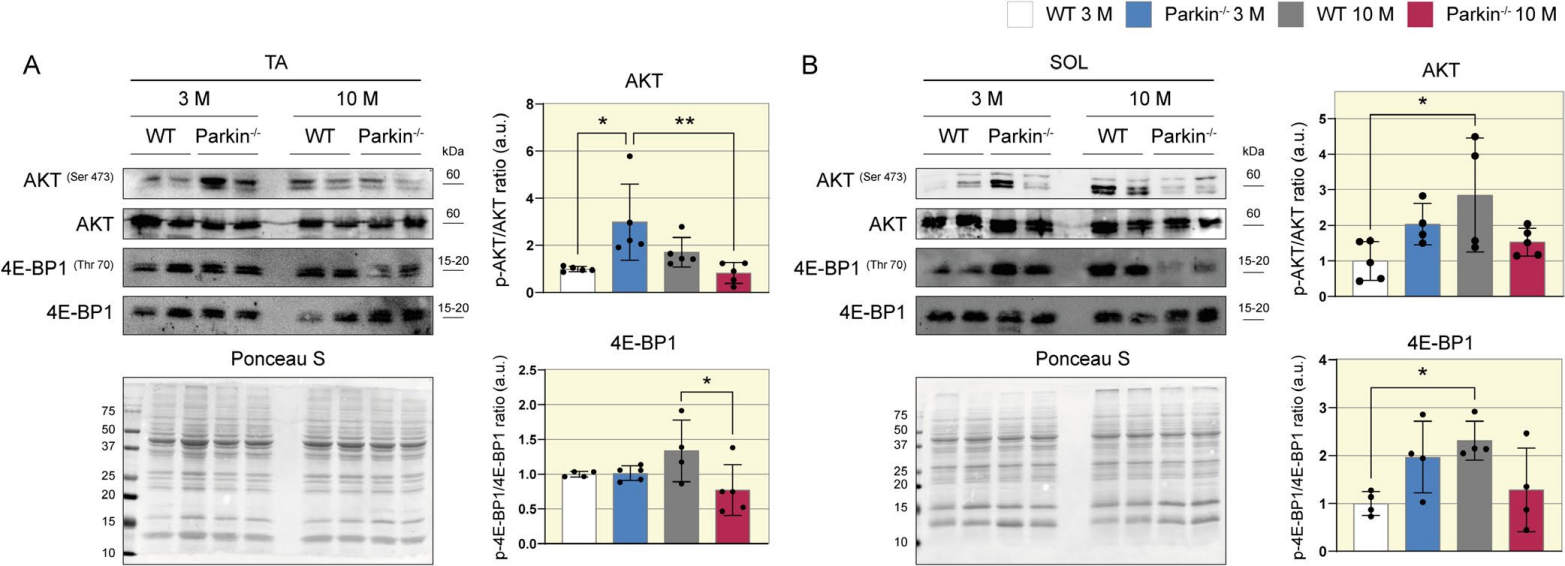
Assessment of protein synthesis in WT and Parkin^−/−^ mice at ages 3 and 10 months. (A) Representative Western Blot images of AKT^(ser473)^, AKT, 4E‐BP1^(Thr70)^, and 4E‐BP1 in TA muscle of WT and Parkin^−/−^ mice at ages 3 and 10 months, with densitometric analysis graphics (*n* = 4–5). Data are presented as means ± SD. One‐way ANOVA followed by Bonferroni's post hoc test: *p < 0.05, **p < 0.01. WT 3 M is set as 1. (B) Representative Western Blot images of AKT^(ser473)^, AKT, 4E‐BP1^(Thr70)^, and 4E‐BP1 in SOL muscle in WT and Parkin^−/−^ mice at ages 3 and 10 months, with densitometric analysis graphics (*n* = 4–5). Data are presented as means ± SD. One‐way ANOVA followed by Bonferroni's post hoc test: *p < 0.05. WT 3 M is set as 1.

**FIGURE 4:**
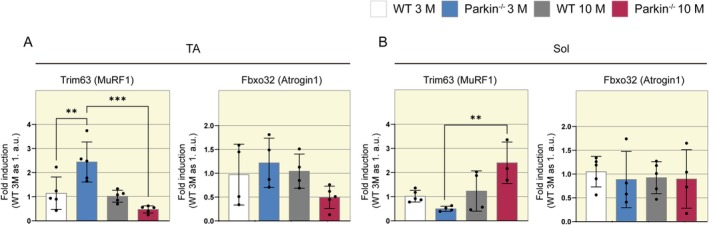
Assessment of proteasomal degradation in WT and Parkin^−/−^ mice at ages 3 and 10 months. (A) Quantitative analysis of Atrogin1 and MuRF‐1 gene expression through qPCR in TA muscle samples from WT and Parkin^−/−^ mice at ages 3 and 10 months (*n* = 4–5). Data are presented as means ± SD. One‐way ANOVA followed by Bonferroni's post hoc test: **p < 0.01, ****p < 0.0001. WT 3 M is set as 1. (B) Quantitative analysis of Atrogin1 and MuRF‐1 gene expression through qPCR in SOL muscle samples from WT and Parkin^−/−^ mice at ages 3 and 10 months (*n* = 3–5). Data are presented as means ± SD. One‐way ANOVA followed by Bonferroni's post hoc test: **p < 0.01. WT 3 M is set as 1.

The atrophic process of skeletal muscle occurs through decreased protein synthesis and increased protein degradation. We observed that the SOL muscle exhibits loss of mass and myofibers diameter in Parkin^−/−^ middle‐aged mice. To investigate the molecular mechanism, we analyzed the level of factors related to protein synthesis in the collected muscles. We assessed the central synthesis pathway activation through phosphorylation of AKT (Ser473) and 4E‐BP1 (Thr70). Interestingly, Parkin^−/−^ mice at 3 months showed higher AKT phosphorylation levels in the TA muscle than the WT in the same age (WT 3 M 1 vs. Parkin^−/−^ 3 M 3 a.u., Figure 3A). However, at 10 months, the phosphorylation level decreased compared to young animals (Parkin^−/−^ 3 M 3 vs. Parkin^−/−^ 10 M 1 a.u., Figure 3A). Additionally, we observed a decrease in 4E‐BP1 phosphorylation in Parkin^−/−^ mice in middle age (WT 10 M 1.2 vs. Parkin^−/−^ 10 M 0.8 a.u., Figure 3A). In the SOL muscle, we observed an increase in the phosphorylation levels of AKT in WT mice at 10 months. (WT 3 M 1 vs. WT 10 M 2.8 a.u., Figure 3B) and 4E‐BP1 (WT 3 M 1 vs. WT 10 M 2.5 a.u., Figure 3B) However, Parkin^−/−^ mice do not show changes. Our findings show that protein synthesis can be modulated differently in Parkin^−/−^ mice and these effects depend on myofiber metabolism.

### 3.5 Increased Expression of *MURF‐1* in the Soleus Muscle of Middle‐Aged Parkin^−/−^ Mice

Since the SOL muscle presents atrophy in type I fibers and activation impairment in pathways related to protein synthesis, we also aimed to investigate genes related to protein degradation. Therefore, we performed gene expression analysis of the *MURF‐1* and *Atrogin‐1*. The TA muscle of Parkin^−/−^ mice showed higher *MURF‐1* expression at 3 months compared to WT mice at the same age (WT 3 M 1 vs. Parkin^−/−^ 3 M 2.5 a.u., Figure 4A), which decreased in middle age (Parkin^−/−^ 3 M 2.5 vs. Parkin^−/−^ 10 M 0.5 a.u., Figure 4A). In contrast, in the SOL muscle, Parkin^−/−^ mice exhibited increased *MURF‐1* expression in the SOL muscle at 10 months (Parkin^−/−^ 3 M 0.5 vs. Parkin^−/−^ 10 M 2.5 a.u., Figure 4B). No significant disparities in *Atrogin‐1* expression were noted across both muscle types and genotypes (Figure 4A,B).

### 3.6 The Soleus Muscle of Parkin^−/−^ Mice at Middle Age Shows a Decrease in Ubiquitin K63 Levels

**FIGURE 5:**
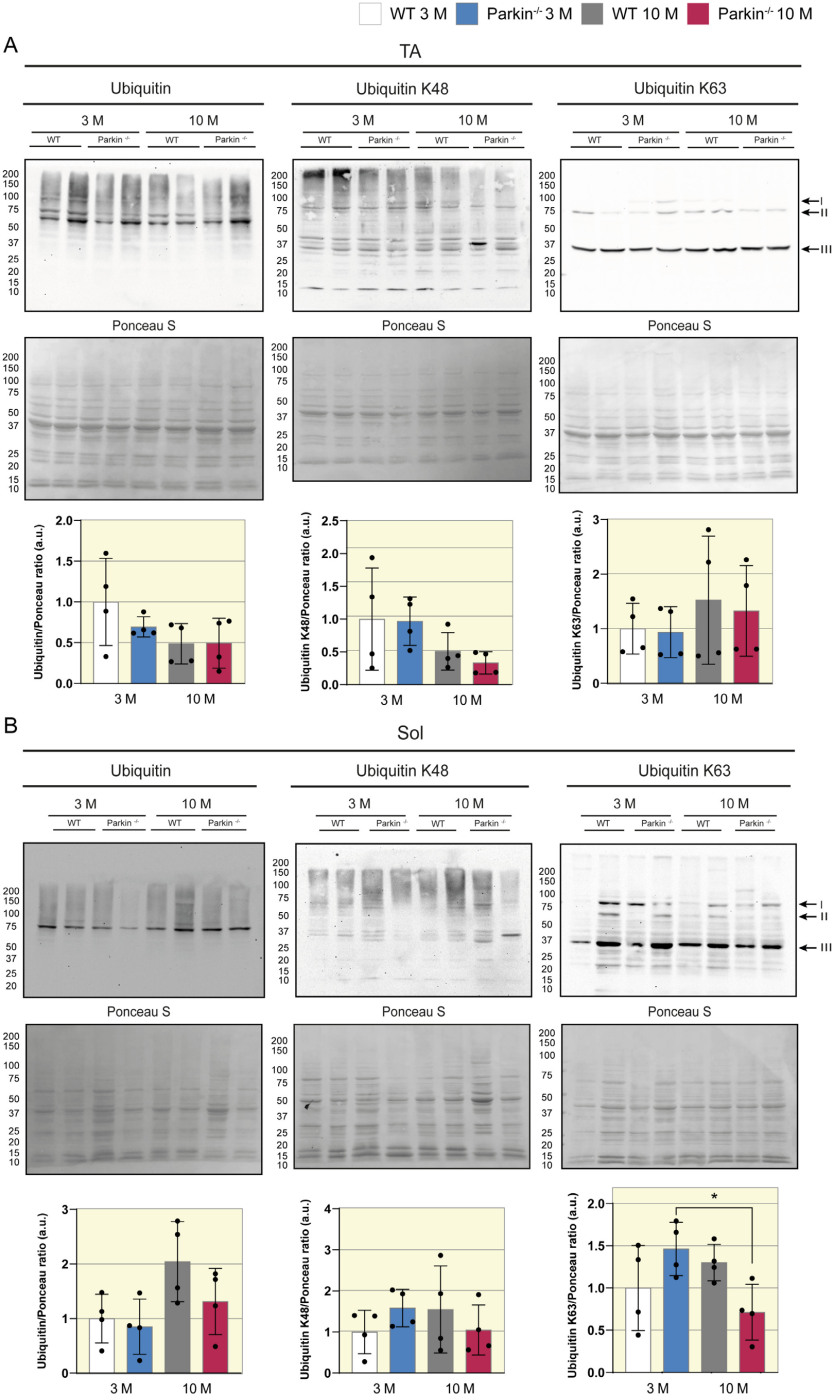
Assessment of ubiquitin‐proteasomal degradation in TA and SOL muscle in WT and Parkin^−/−^ mice at 3 and 10 months. (A) Representative Western Blot images of Ubiquitin, Ubiquitin K48, and Ubiquitin K63 in TA muscle of WT and Parkin^−/−^ mice at ages 3 and 10 months, with densitometric analysis graphics (*n* = 4). Data are presented as means ± SD. WT 3 M is set as 1. (B) Representative Western blot images of Ubiquitin, Ubiquitin K48, and Ubiquitin K63 in SOL muscle of WT and Parkin^−/−^ mice at ages 3 and 10 months, with densitometric analysis graphics (*n* = 4). Data are presented as means ± SD. One‐way ANOVA followed by Bonferroni's post hoc test: *p < 0.05. WT 3 M is set as 1.

Given the significant differences observed in *MURF‐1*, which is associated with protein degradation, we decided to further investigate proteins related to proteasomal and autophagic pathways. We analyzed the protein content of Ubiquitin, Ubiquitin K63, and Ubiquitin K48. We did not observe differences between the groups in the TA muscle (Figure 5A). However, the SOL muscle of Parkin^−/−^ mice has a decrease in the Ubiquitin K63 content in middle age, compared to the same genotype at 3 months (Parkin^−/−^ 3 M 1.4 vs. Parkin^−/−^ 10 M 0.8 a.u., Figure 5B). Also, we observed that the SOL muscle of Parkin^−/−^ mice had a lower LC3I protein content at 3 months (WT 3 M 1 vs. Parkin^−/−^ 3 M 0.5 a.u., Figure S1D) and a lower LC3II/I ratio at 10 months (WT 10 M 1.9 vs. Parkin^−/−^ 10 M 0.6 a.u., Figure S1D). These results show that at specific loss of oxidative myofibers, the SOL muscle in the absence of Parkin may present impairments in ubiquitin‐proteasome and autophagy‐lysosomal pathways.

### 3.7 Middle‐Aged Parkin^−/−^ Mice Exhibit No Differences in Mitochondrial Protein Levels

**FIGURE 6:**
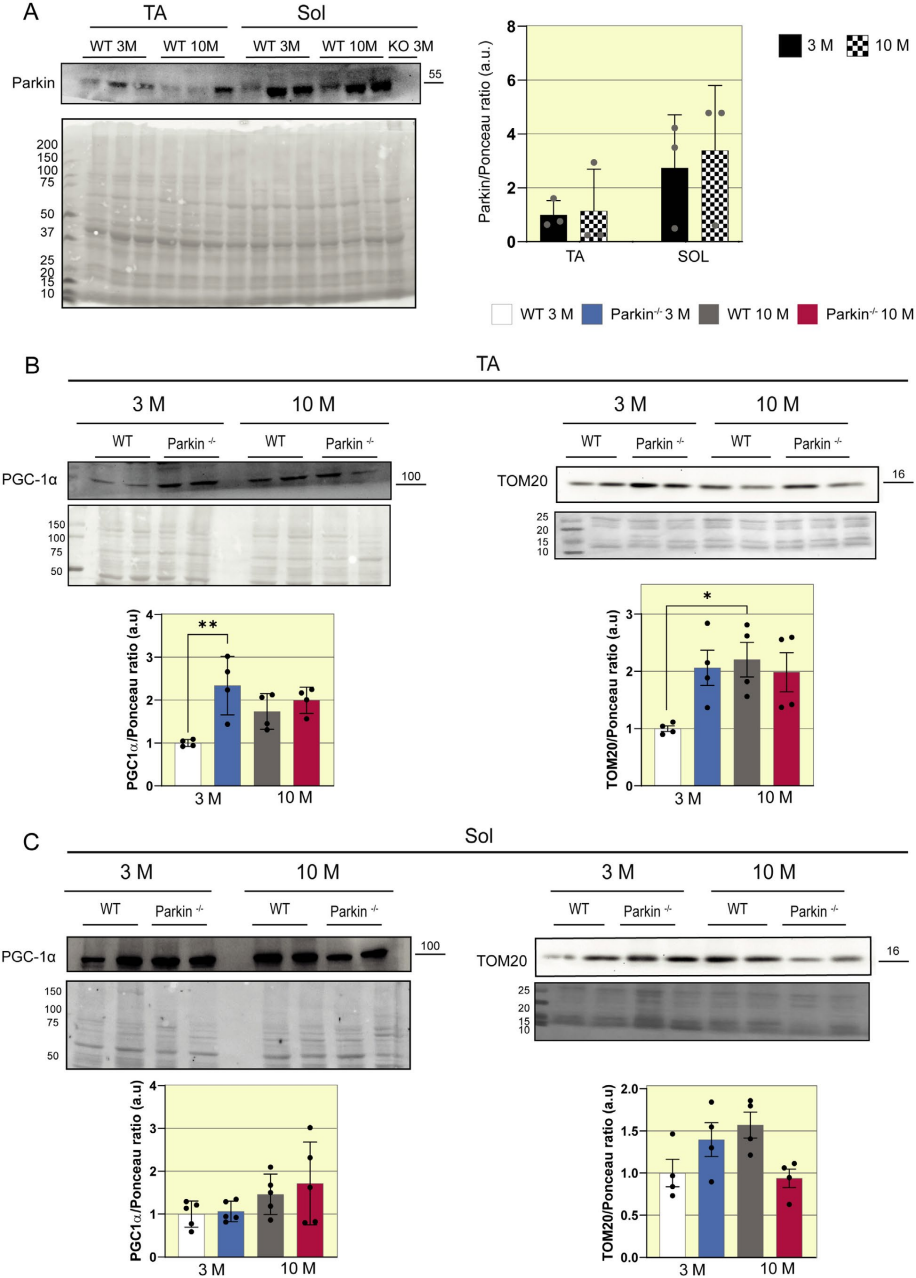
Assessment of mitochondrial markers in SOL and TA muscle in WT and Parkin^−/−^ mice at 3 and 10 months. (A) Representative Western blot image of Parkin in TA and SOL muscles of WT mice at ages 3 and 10 months, with densitometric analysis graphics (*n* = 3). Data are presented as means ± SD. (B) Representative Western blot images of PGC‐1α and Tom20 in TA muscle in WT and Parkin^−/−^ mice at ages 3 and 10 months, with densitometric analysis graphics (*n* = 4). Data are presented as means ± SD. One‐way ANOVA followed by Bonferroni's post hoc test: *p < 0.05, **p < 0.01. WT 3 M is set as 1. (C) Representative Western blot images of PGC‐1α and Tom20 in SOL muscle in WT and Parkin^−/−^ mice at ages 3 and 10 months, with densitometric analysis graphics (*n* = 4–5). Data are presented as means ± SD. WT 3 M is set as 1.

The efficiency of protein degradation mediated by the ubiquitin‐proteasome system entails a substantial ATP demand, which is tightly linked to mitochondrial functional integrity [23]. Furthermore, over 60% of the mitochondrial proteome is susceptible to ubiquitination, indicating a robust functional crosstalk between mitochondrial dynamics and the ubiquitin‐proteasome system [24]. We analyzed the content of Parkin, TOM20 (Translocase of the outer membrane 20), a mitochondrial transport membrane protein, and PGC‐1α (peroxisome proliferator‐activated receptor gamma coactivator‐1 alpha), an important marker of mitochondrial biogenesis. In Parkin protein content, we do not observe statistical differences between groups. In the TA muscle, Parkin^−/−^ mice at 3 months of age show higher levels of PGC‐1α than WT mice at the same age, which is lost at middle age (WT 3 M 1 vs. Parkin^−/−^ 3 M 2.5 a.u., Figure 6B). In the analysis of TOM20, its content increased in the TA muscle of WT mice at middle age, compared to the same genotype at 3 months (WT 3 M 1 vs. WT 10 M 2.2 a.u., Figure 6B). We did not observe significant differences in PGC‐1α and TOM20 in the SOL muscle (Figure 6C).

### 3.8 The Soleus Muscle of Parkin^−/−^ Mice Exhibits Impairment in the Mitochondrial Respiratory Chain

**FIGURE 7:**
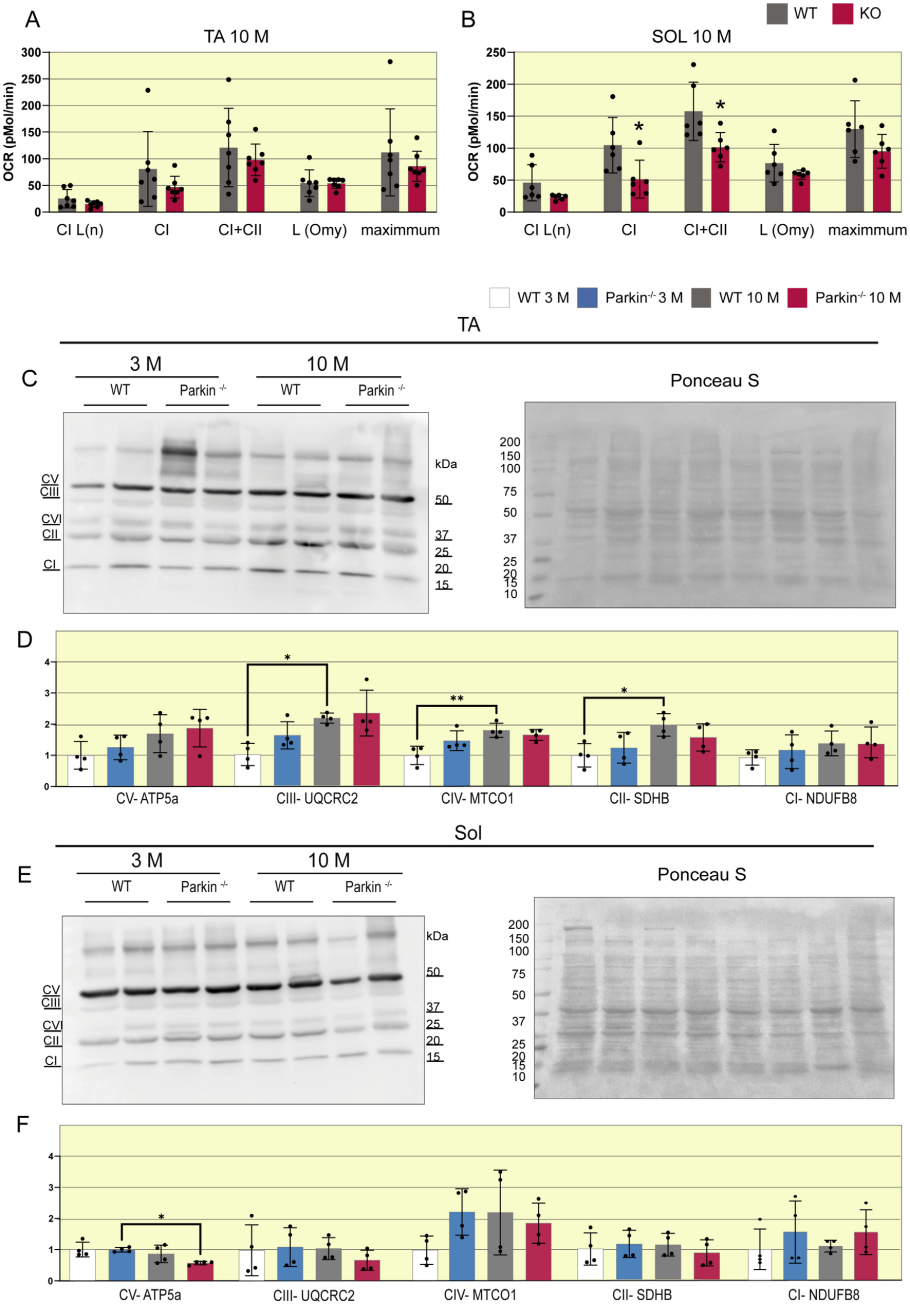
High‐resolution respirometry and Assessment of mitochondrial markers in SOL and TA muscle in WT and Parkin^−/−^ mice at 10 months. (A) High‐resolution respirometry was used to measure the oxygen consumption rate (OCR) in TA muscle samples from WT and Parkin^−/−^ mice at 10 months of age (*n* = 7). Data are presented as means ± SD. WT 10 M is set as 1. (B) High‐resolution respirometry was used to measure the oxygen consumption rate (OCR) in SOL samples from WT and Parkin^−/−^ mice at age 10 months (*n* = 6). Data are presented as means ± SD. Student's *t*‐test: *p < 0.05. WT 10 M is set as 1. (C) Representative Western blot images of ATP5a, UQCRC2, MTCO1, SDHB, and NDUFB8 in TA muscle samples from WT and Parkin^−/−^ mice at 3 and 10 months of age. (D) Graphical representations of the protein content of OXPHOS complexes in TA muscle. Data are presented as mean ± SD. The solid dash represents One‐way ANOVA followed by Bonferroni's post hoc test: *p < 0.05, **p < 0.01. (E) Representative Western blot images of ATP5a, UQCRC2, MTCO1, SDHB, and NDUFB8 in SOL muscle samples from WT and Parkin^−/−^. (F) Graphical representations of the protein content of OXPHOS complexes in SOL muscle. Data are presented as mean ± SD. The solid dash represents One‐way ANOVA followed by Bonferroni's post hoc test: *p < 0.05.

Mitochondrial respiratory capacity is directly related to muscle mass control and atrogene expression [25]. Therefore, our last objective was to analyze the oxygen consumption of the TA and SOL muscles in middle‐aged WT and Parkin^−/−^ mice. For this purpose, the muscles were extracted and analyzed using the Oroboros O2k equipment. Our findings demonstrate that the TA did not undergo significant alterations (Figure 7A). However, the SOL muscle of Parkin^−/−^ mice showed negative regulation in two complexes of the respiratory chain: Complex II (WT 10 M 100 vs. Parkin^−/−^ 10 M 50 pMol/min, Figure 7B) and maximum respiration (WT 10 M 160 vs. Parkin^−/−^ 10 M 100 pMol/min, Figure 7B). Since we observed significant changes in mitochondrial respiration in the SOL muscle, we analyzed the protein content of the OXPHOS complex. The TA muscle of middle‐aged WT mice shows an increase in Complex II (SDHB) (WT 3 M 1 vs. WT 10 M 2 a.u., Figure 7D), Complex III (UQCRC2) (WT 3 M 1 vs. WT 10 M 2.2 a.u., Figure 7D) and Complex IV (MTCO1) (WT 3 M 1 vs. WT 10 M 1.8 a.u., Figure 7D) compared to the same genotype at 3 months of age. In the SOL muscle, the absence of Parkin leads to a decrease just in Complex V (ATP5a) (Parkin^−/−^ 3 M 1 vs. Parkin^−/−^10 M 0.5 a.u., Figure 7F) in middle‐aged mice, compared to the same genotype more young. Together, these results suggest that the absence of Parkin impairs age‐associated adaptations in OXPHOS protein expression, particularly in the SOL muscle, where reduced ATP5a levels align with decreased respiratory capacity in midlife.

## 4 Discussion

The progression of Parkinson's disease has been associated with skeletal muscle mass loss. The sarcopenic process arising from the condition may be linked to various mechanisms, including neurodegeneration, immobility, malnutrition, and even advanced age [26]. Our study is the first to investigate changes in the skeletal muscle of an animal model of early Parkinson's disease before the appearance of motor symptoms arising from the condition. We observed that the loss of Parkin leads to a reduction in maximum isometric force generation, a decrease in the size of oxidative myofibers, and molecular alterations in protein synthesis and degradation pathways in both glycolytic and oxidative myofibers in midlife. Until now, there has been no evidence of how the absence of Parkin affected muscle tissue in different fibers at different ages. We also demonstrated that the motor capacity of the animals is not affected despite the histological and molecular modifications in skeletal muscle.

We used the Walking Beam Test to assess motor coordination and balance in our experimental model. This test can detect motor deficits related to aging, brain injuries, and pharmacological effects of certain drugs [17]. A Parkin mutant mouse model (ParkinS65A/S65A) previously exhibited balance problems in a similar test, even before neurodegeneration [27]. Our study observed that Parkin^−/−^ mice in middle age did not show deficits in balance but required more steps to traverse the narrow beam, representing a more challenging task. Additionally, we applied the Open Field Test to analyze the mice's motor locomotion. This test evaluates locomotion and analyzes anxiety‐related parameters, such as exploring different quadrants. When placed in the center of the apparatus, animals typically run toward the edges and explore their surroundings. As they become habituated to the environment, anxiety decreases, and they tend to spend more time in the central regions of the field [28]. Our results indicate greater ambulation of Parkin^−/−^ mice at 10 months compared to the same genotype and WT mice.

Furthermore, at 3 months of age, WT mice have greater ambulation than Parkin^−/−^ mice; however, there is a decrease in middle age. When measuring ambulation in the central area, there is a significant increase in the Parkin^−/−^ genotype in middle age, compared to the same genotype in young mice, which helps us to answer that the absence of Parkin does not seem to generate signs of anxiety in mice (Figure S1A). In this way, our findings align with previous literature since several studies have shown Parkin knockout animal models did not demonstrate impairment in locomotion in the Open Field Test [29, 30]. We have also performed tests to measure muscular aerobic power and physical resistance through the Maximum Power Test on a treadmill and the Spider Mice Test. Previously, Chen et al. [31] used Parkin^−/−^ mice in a voluntary running exercise protocol and found no differences in performance compared to the wild‐type group. In this way, in our study, both genotypes exhibited comparable aerobic power and endurance in middle age. Under the Spider Mice protocol, both genotypes in midlife showed a reduction in the time spent hanging. One possible explanation is that total body mass may impact this test, as both genotypes demonstrated increased body mass. Additionally, we conducted an analysis of specific force generation in isolated fibers from the FDB muscle. In a previous study, Gouspillou et al. [12] reported a reduction in the specific strength of the TA muscle in Parkin^−/−^ mice during in situ assessments. Our results align with these findings since we observed a decline in isometric force in middle‐aged Parkin^−/−^ mice when compared to WT mice. In summary, our data suggest that the absence of Parkin leads to a reduction of specific force generation in middle‐aged mice.

Our initial observations indicated a reduction in SOL muscle mass in middle‐aged Parkin^−/−^ mice. Intriguingly, the TA muscle exhibited no discernible differences. Furthermore, our histological analysis unveiled a concurrent decrease in the diameter of type I myofibers, predominantly oxidative, in the SOL muscle in the absence of Parkin in middle‐aged mice. In contrast, the TA muscle, composed predominantly of glycolytic myofibers, remained unchanged. Skeletal muscle atrophy affects fiber types differently depending on the stimulus, such as muscle disuse and spinal cord injuries decreasing the diameter of oxidative myofibers. At the same time, cachexia and aging predominantly affect glycolytic myofibers [32]. In Parkin^−/−^ mice, Gouspillou et al. [12] demonstrated a propensity for type IIb myofibers (glycolytic predominance) to exhibit a larger cross‐sectional area (CSA) when compared to control mice. However, the impact of Parkin's absence on various muscle fiber types in middle age has yet to be previously documented. Our findings indicate that middle‐aged Parkin^−/−^ mice show impairment mainly in oxidative myofibers. Unlike an initial sarcopenic model, where myofibers with a glycolytic predominance are predominantly affected, our study aligns with the atrophy observed in the muscle disuse model [33].

The maintenance of skeletal muscle mass occurs through a balance between protein synthesis and degradation [34]. To analyze protein synthesis, we used Western blotting to measure the levels of AKT and 4E‐BP1 proteins. AKT is a serine/threonine kinase that mediates cell growth and survival in various tissues and promotes protein synthesis in multiple ways [35]. The literature provides evidence that phosphorylation of AKT at Ser 473 decreased in skeletal muscle of aged mice and increased during physical exercise, showing the important role of protein in regulating muscle mass [36, 37]. Although we observed a decline in AKT phosphorylation at TA muscle in middle‐aged Parkin^−/−^ mice, this reduction is compared to the same genotype at 3 months of age, with significantly higher protein activity. The SOL muscle does not exhibit lower AKT phosphorylation in the Parkin^−/−^ mice at 3 and 10 months. However, it does not parallel the heightened activation seen in WT mice in middle age, suggesting an impairment in this pathway in knockout mice.

Phosphorylation of 4E‐BP1 leads to its dissociation from eIF4E, allowing the binding of eIF4E to eIF4G, which initiates translation [38]. In skeletal muscle tissue, 4E‐BP1 signaling is also essential for maintaining homeostasis, protecting against dysfunctions associated with aging and obesity, and playing a critical role in the neuromuscular junction [39]. In the 4E‐BP1 analysis, in TA muscle, curiously we observed a decrease in phosphorylation in Parkin^−/−^ mice middle‐aged, when compared to WT mice middle‐aged. In SOL muscle, we observed an increase in 4E‐BP1 phosphorylation in WT mice middle‐aged, compared to WT mice at 3 months, but Parkin^−/−^ mice middle‐aged do not show this modulation. This is not the first time an interaction between Parkin and protein related to the protein synthesis pathway has been demonstrated. Under conditions of mitochondrial stress in HeLa cells, mTORC1 is ubiquitinated by Parkin through ubiquitination sites K2066 and K2306, preventing the downregulation of this pathway and promoting cell survival [40]. Another study showed that Parkin overexpression in skeletal muscle of young mice promoted hypertrophy and increased AKT/mTORC1 pathway activity. Furthermore, in elderly mice, Parkin overexpression protects against age‐related muscle loss [15]. Considering Parkin's ability to interact with proteins related to protein synthesis, a possible explanation for our results is that the absence of Parkin leads to a decrease in protein synthesis in middle‐aged mice in both muscles, but only in the SOL muscle does this modulation may be related to muscle atrophy.

Since the balance between protein synthesis and degradation is critical to skeletal muscle, we analyze the expression of the *Atrogin‐1* and *MURF‐1* genes. Atrogin‐1 and MURF‐1 are E3 ubiquitin ligases that target proteins in the proteasome system through polyubiquitination [41]. Atrogin‐1 degrades eIF3‐F, a critical translation initiation factor in eukaryotic cells, while *MURF‐1* targets contractile proteins of the myosin chain [42, 43]. Our findings showed increased expression of *MURF‐1* in the SOL muscle of Parkin^−/−^ mice in middle age, whereas no such increase was observed in the WT mice. In the TA muscle, interestingly, we observed a decreased expression of *MURF‐1* in the Parkin^−/−^ genotype in middle age. Prior studies have demonstrated elevated levels of *MURF‐1* in the absence of Parkin. Ito et al. [44] reported an augmentation in protein levels in skeletal muscle, particularly *MURF‐1*, in Parkin^−/−^ mice subjected to cigarette smoke exposure. In a separate investigation, Peker et al. [14] induced prolonged fasting in Parkin^−/−^ mice, observing heightened *MURF‐1* activity in the gastrocnemius muscle, although no alterations were noted at baseline. The referenced studies documented alterations in *MURF‐1* within animal models subjected to specific interventions. Our study, in contrast, represents the first to observe heightened *MURF‐1* expression in Parkin^−/−^ mice during the aging process. Moreover, our findings highlight that this upregulation seems more relevant to oxidative muscles.

The indirect connection regarding AKT activity and *MURF‐1* expression may explain our findings through a “super‐compensation” system. The TA muscle, in the absence of Parkin, shows high expression levels of AKT and *MURF‐1* at 3 months of age, simultaneously, and at 10 months. Despite the decrease in protein synthesis signaling, it does not undergo atrophy due to the reduction of *MURF‐1* expression. On the other hand, the SOL muscle, which showed reduced myofiber diameter in Parkin^−/−^ mice in middle age, does not experience a decrease in protein synthesis, but at 10 months, the Parkin^−/−^ mice increases degradation without a corresponding increase in protein synthesis signaling, resulting in an unbalanced protein turnover. One unanswered question in our study is about mTOR protein levels. mTOR is a key factor for protein synthesis, but we did not have the necessary antibody for analysis. Another limitation of our study was that we decided only to perform gene expression of the atrogenes MURF‐1 and Atrogin. The mouse SOL muscle is tiny from further analysis by performing other experiments.

Furthermore, our results obtained in the analyses of ubiquitin and autophagic proteins may be related to increase in *MURF‐1* expression. It is known that inhibition of autophagy leads to muscle atrophy and that *MURF‐1* is increased in skeletal muscle in rodent models with deficiency in autophagic flux, such as Atg7^−/−^ and Atg5^−/−^ mice [45]. In our study, the SOL muscle of Parkin^−/−^ mice has lower levels of LC3I at 3 months of age, and the levels remain the same at middle age. In the same muscle, we also observed a decrease in the LC3II/I ratio in the absence of Parkin, at middle age. It is important to emphasize that we did not observe significant differences in the p62 protein (Figure S1D), and that other techniques would be necessary to measure the autophagic flux. Also, autophagy plays a critical role in the degradation of misfolded or damaged proteins [46]. Studies show that Parkin‐mediated Lys63‐linked polyubiquitination sequesters misfolded proteins and mediates the release of these proteins for autophagy [47]. Our findings indicate that the SOL muscle of Parkin^−/−^ mice has a decrease in Ubiquitin K63 content in middle age. This finding together with the observed loss of SOL muscle mass suggests an impairment in the ubiquitination protein control in the absence of Parkin during the middle age.

Studies show that atrophy resulting from prolonged inactivity induces mitochondrial dysfunction, reducing their respiratory capacity and coupling [48]. Previously, a study showed that in C2C12 myotubes, the Parkin knockdown showed impairments in mitochondrial turnover and atrophy in vitro [13]. Therefore, we investigated proteins related to mitochondrial quality control. PGC‐1α is considered the primary regulator of mitochondrial biogenesis, and in atrophic muscle, a decrease in its content is associated with dysfunction in the mitochondrial respiratory chain [49]. Our results show that the TA muscle of Parkin^−/−^ mice has higher PGC‐1α levels at 3 months compared to the WT genotype, and both genotypes do not have changes in middle age. However, no differences in PGC‐1α content were observed between groups in the SOL muscle, suggesting that the atrophy detected in this muscle may not be directly associated with impaired mitochondrial biogenesis. Also, we analyzed the protein content of TOM20. After mitochondrial depolarization, Parkin mediates degradation of some outer membrane proteins such as TOM20 [50]. We observed no differences in TOM20 levels between groups at middle age, suggesting that mitochondrial mass is preserved.

To further investigate mitochondrial function, we assessed mitochondrial respiration using high‐resolution respirometry and evaluated OXPHOS protein levels. We found a difference in the SOL muscle of Parkin^−/−^ animals in middle age, which exhibited lower respiration in the presence of succinate (Complex II) and ADP (OXPHOS capacity). Additionally, ATP5a (Complex V) levels were reduced in middle‐aged Parkin^−/−^ mice compared to the same genotype at 3 months of age. Notably, no differences were observed between genotypes in the TA muscle at middle age, suggesting that this impairment is specific to the SOL muscle.

The literature regarding Parkin's role in mitochondrial respiration remains inconclusive. A recent study reported that the soleus and gastrocnemius muscles of Parkin^−/−^ mice exhibit normal OXPHOS function and mtDNA content at 15 months of age [51]. However, that study did not include younger mice and employed a different Parkin knockout model from the one used in our study. In contrast, another study analyzed leukocytes from patients with Parkin mutations and found reduced complex I activity [52]. Similarly, in a Drosophila model of neurotoxicity associated with amyotrophic lateral sclerosis, Parkin overexpression was shown to restore complex I and III activities [53]. These findings support a direct role for Parkin in mitochondrial respiration and are in line with our current results. The SOL muscle, composed primarily of oxidative fibers, is particularly affected by the absence of Parkin in middle age, showing type I fiber atrophy, increased protein degradation, and impaired mitochondrial respiration and complex V activity. Interestingly, the TA muscle does not exhibit these alterations, suggesting that, likely due to its higher mitochondrial demand, the SOL muscle relies more heavily on Parkin activity.

In conclusion, our findings highlight a muscle‐specific role of Parkin in maintaining mitochondrial integrity and metabolic homeostasis during aging, particularly in the SOL muscle. We present a summary of our key findings in the graphical abstract. Our findings contribute to a better understanding of the role of Parkin in skeletal muscle and provide new insights into the maintenance of skeletal muscle mass during aging.

## Author Contributions

**Isabela Aparecida Divino:** conceptualization, investigation, visualization, methodology, formal analysis, data curation, writing – original draft. **Ana Laura da Vieira‐da‐Silva:** formal analysis, investigation, visualization, writing – review and editing. **Marcos Vinicius Esteca:** conceptualization, data curation, methodology, writing – review and editing. **Rafael Paschini Tonon:** investigation, methodology, writing – review and editing. **Felipe Oliveira Gomes da Cruz:** investigation, methodology, writing – review and editing. **Renata Rosseto Braga:** investigation, methodology, visualization. **Eduardo Rochete Ropelle:** resources, supervision, writing – review and editing. **Paulo Guimarães Gandra:** resources, writing – original draft, writing – review and editing. **Igor Luchini Baptista:** conceptualization, project administration, visualization, supervision, funding acquisition, writing – original draft, writing – review and editing.

## Conflicts of Interest

The authors declare no conflicts of interest.

## Supporting information


**Figure S1.** (A) Open Field Test (Area central) of WT and Parkin^−/−^ mice at ages 3 and 10 months. The graphic demonstrates the analysis of ambulation in the area central (*n* = 6–10). Data are presented as means ± SD. One‐way ANOVA followed by Bonferroni’s post hoc test: *p < 0.05. WT 3 M is set as 1. (B) Time to fatigue data from flexor digitorum brevis (FDB) muscle fibers from WT and Parkin^−/−^ mice (*n* = 5 fibers from 3 WT mice and *n* = 5 fibers from 3 and Parkin^−/−^ mice). Data are presented as means ± SEM. Two‐way ANOVA followed by Bonferroni’s post hoc test. (C) Representative Western blot images of LC3‐I and p62 in tibial muscle (TA) in WT and Parkin^−/−^ mice at ages 3 and 10 months, with densitometric analysis graphics (*n* = 4). Data are presented as means ± SD. WT 3 M is set as 1. (D) Representative Western blot images of LC3‐I/II and p62 in soleus muscle (SOL) in WT and Parkin^−/−^ mice at ages 3 and 10 months, with densitometric analysis graphics (*n* = 4). Data are presented as means ± SD. One‐way ANOVA followed by Bonferroni’s post hoc test: *p < 0.05, **p < 0.01. WT 3 M is set as 1.
**Table S1.** Primary antibodies.
**Table S2.** Secondary antibodies.
**Table S3.** Primers.

## Data Availability

The data that support the findings will be available in Figshare at https://doi.org/10.6084/m9.figshare.25743000.v1 and available from the corresponding author I.L.B. on request.
