# Supplementary material for: Absence of Parkin Results in Atrophy of Oxidative Myofibers and Modulation of AKT and MURF1 Signaling in Middle‐Aged Male Mice
Source: Acta Physiol (Oxf). 2025 Jul 29;241(9):e70082. doi: 10.1111/apha.70082 (PMC12308108; doi:10.1111/apha.70082)
Supplement: Supplementary file 1 — Figure S1. (A) Open Field Test (Area central) of WT and Parkin−/− mice at ages 3 and 10 months. The graphic demonstrates the analysis of ambulation in the area central (n = 6–10). Data are presented as means ± SD. One‐way ANOVA followed by Bonferroni’s post hoc test: *p < 0.05. WT 3 M is set as 1. (B) Time to fatigue data from flexor digitorum brevis (FDB) muscle fibers from WT and Parkin−/− mice (n = 5 fibers from 3 WT mice and n = 5 fibers from 3 and Parkin−/− mice). Data are presented as means ± SEM. Two‐way ANOVA followed by Bonferroni’s post hoc test. (C) Representative Western blot images of LC3‐I and p62 in tibial muscle (TA) in WT and Parkin−/− mice at ages 3 and 10 months, with densitometric analysis graphics (n = 4). Data are presented as means ± SD. WT 3 M is set as 1. (D) Representative Western blot images of LC3‐I/II and p62 in soleus muscle (SOL) in WT and Parkin−/− mice at ages 3 and 10 months, with densitometric analysis graphics (n = 4). Data are presented as means ± SD. One‐way ANOVA followed by Bonferroni’s post hoc test: *p < 0.05, **p < 0.01. WT 3 M is set as 1. Table S1. Primary antibodies. Table S2. Secondary antibodies. Table S3. Primers. [file APHA-241-e70082-s001.docx]

Table 1. Primary antibodies

| Antibody | Code | Brand | Concentration |
| --- | --- | --- | --- |
| p-4E-BP1 (T70) | 9455s | Cell Signaling | 1:1000 |
| 4E-BP1 | 9452s | Cell Signaling | 1:1000 |
| PGC-1α | Pa5-38022 | Invitrogen | 1:1000 |
| p-AKT (ser473) | sc-33437 | Santa Cruz | 1:500 |
| AKT | sc-8312 | Santa Cruz | 1:500 |
| Ubiquitin | PA1-10023 | Invitrogen | 1:1000 |
| Ubiquitin k48 | MA5-35382 | Invitrogen | 1:1000 |
| Ubiquitin k63 | MA5-32573 | Invitrogen | 1:1000 |
| TOM20 | 42406s | Cell Signaling | 1:1000 |
| LC3A/B | 4108s | Cell Signaling | 1:1000 |
| SQSTM1/p62 | 23214s | Cell Signaling | 1:1000 |
| OXPHOS | ab110413 | Abcam | 1:1000 |

Table 2. Secondary antibodies

| Antibody | Code | Brand | Concentration |
| --- | --- | --- | --- |
| Donkey Anti-Goat Polyclonal | 705-035-003 | Jackson | 1:10000 |
| Goat Anti-Rabbit Polyclonal | 111-035-003 | Jackson | 1:10000 |

Table 3 . Primers

| Gene | Forward | Reverse |
| --- | --- | --- |
| MuRF-1 | GTGTGAGGTGCCTACTTGCT | ACTCAGCTCCTCCTTCACCT |
| Mafbx (Atrogin 1) | TACTAAGGAGCGCCATGGATACT | GTTGAATCCTCTGGAATCCAGGAT |
| GAPDH | CATCACTGCCACCCAGAAGACTG | ATGCCAGTGAGCTTCCCGTTCAG |

**Supplementary Figure 1. (A)** Open Field Test (Area central) of WT and Parkin^-/-^ mice at ages 3 and 10 months. The graphic demonstrates the analysis of ambulation in the area central (n=6-10). Data are presented as means ± SD. One-way ANOVA followed by Bonferroni's post hoc test: **p* < 0.05. WT 3 M is set as 1. (B) Time to fatigue data from flexor digitorum brevis (FDB) muscle fibers from WT and Parkin^-/-^ mice (n = 5 fibers from 3 WT mice and n = 5 fibers from 3 and Parkin^-/-^ mice). Data are presented as means ± SEM. Two-way ANOVA followed by Bonferroni's post hoc test. **(C)** Representative Western Blot images of LC3-I and p62 in tibial muscle (TA) in WT and Parkin^-/-^ mice at ages 3 and 10 months, with densitometric analysis graphics (n=4). Data are presented as means ± SD. WT 3 M is set as 1. **(D)** Representative Western Blot images of LC3-I/II and p62 in soleus muscle (SOL) in WT and Parkin^-/-^ mice at ages 3 and 10 months, with densitometric analysis graphics (n=4). Data are presented as means ± SD. One-way ANOVA followed by Bonferroni's post hoc test: **p* < 0.05, ***p* < 0.01. WT 3 M is set as 1.
